# Competitions for Hospital Buildings: Further Opinions

**Published:** 1912-03-16

**Authors:** William Henman

**Affiliations:** 19 Temple Street, Birmingham


### THE EDITOR'S LETTER BOX.

### Competitions for Hospital Buildings: further opinions.

*To the Editor of* The Hospital.

Sir,—The articles in your issue of the 2nd inst. are both opportune and important, because awards in recent competitions have undoubtedly displayed a total disregard of the practical work for which hospitals should be designed. Unless, therefore, assessors are appointed with intimate and practical knowledge of requirements, combined with the strictest impartiality and firmness to resist pressure from importunate promoters, public interests must suffer by the selection of designs which are defective in important particulars.

Such, unfortunately, has happened both at Bradford and at Hastings. In the former case it is significant that further progress is deferred, and in the latter it is to be hoped a similar decision may be come to.

After careful study of the published plans, I wrote to the Chairman and Committee. A copy of the letter is sent herewith. Its publication may be enlightening and tend to prevent waste of money and further disappointment.

To invariably accept an assessor's award without complaint may appear magnanimous to Messrs. Adams and Holden, but is it in the true interests of the public or of the architectural profession? Their plans are infinitely superior to those placed 1 and 2, and are quite workable in almost every particular. They also practically comply with the conditions of competition (except, perhaps, as regards cost), which, however, were not drawn up in a manner to secure the best result.—Yours truly,

William Henman, F.R.I.B.A.

19 Temple Street, Birmingham,
March 11.

[The enclosure is too long for us to publish this week.—Ed., The Hospital.]

